# Development and Validation of ScriptTaq COVID PCR: An In-House Multiplex rRT-PCR for Low-Cost Detection

**DOI:** 10.3390/cimb44120417

**Published:** 2022-12-05

**Authors:** Dana Abdalghani AbuObead, Tasnim Khalid Alhomsi, Mahmoud Zhra, Bandar Alosaimi, Muaawia Hamza, Maaweya Awadalla, Osama Ezzeldin Abdelhadi, Joud Abdullah Alsharif, Liliane Okdah, Khaled AlKattan, Saeed Al Turki, Hana M. A. Fakhoury, Ahmad Aljada

**Affiliations:** 1 College of Medicine, Alfaisal University, Riyadh 11533, Saudi Arabia; 2 Research Center, King Fahad Medical City, Riyadh Second Health Cluster, Riyadh 11525, Saudi Arabia; 3 Faculty of Medicine, King Fahad Medical City, Riyadh 11525, Saudi Arabia; 4 Infectious Disease Research Department, King Abdullah International Medical Research Center, Riyadh 11481, Saudi Arabia; 5 Anwa Medical Labs, King Saud University, Riyadh 11451, Saudi Arabia; 6 Department of Biochemistry and Molecular Medicine, College of Medicine, Alfaisal University, Riyadh 11533, Saudi Arabia

**Keywords:** COVID-19, SARS-CoV-2, multiplex RT-PCR, in-house RNA isolation, silica-based RNA isolation, magnetic beads RNA isolation

## Abstract

The COVID-19 pandemic necessitated an extensive testing for active SARS-CoV-2 infection. However, securing affordable diagnostic tests is a struggle for low-resource settings. We report herein the development and validation of an in-house multiplex real-time RT-PCR diagnostic test for the detection of active COVID-19 infection (ScriptTaq COVID PCR). Furthermore, we describe two methods for RNA extraction using either an in-house silica column or silica-coated magnetic beads to replace commercial RNA extraction kits. Different buffer formulations for silica column and silica-coated magnetic beads were tested and used for RNA isolation. Taq polymerase enzyme and thermostable reverse transcriptase enzyme were purified from bacterial clones. Primers/probes sequences published by the WHO and CDC were used for the qualitative detection of the *RNA-dependent RNA polymerase* (*RdRp*) and *nucleocapsid* (*N*) genes, respectively. ScriptTaq COVID PCR assay was able to detect up to 100 copies per reaction of the viral *RdRP* and *N* genes. The test demonstrated an overall agreement of 95.4%, a positive percent agreement (PPA) of 90.2%, and a negative percent agreement (NPA) of 100.0% when compared with two commercially available kits. ScriptTaq COVID PCR diagnostic test is a specific, sensitive, and low-cost alternative for low-resource settings.

## 1. Introduction

Severe acute respiratory syndrome coronavirus 2 (SARS-CoV-2) is a positive-sense single-stranded RNA virus and is the causative agent for coronavirus disease 2019 (COVID-19). The early identification and isolation of infected individuals is pivotal for COVID-19 pandemic control. Currently, diagnosis of acute SARS-CoV-2 infection relies on either viral RNA or viral antigens’ detection. Viral RNA tests detect nucleic acid sequences specific to SARS-CoV-2 by real-time reverse transcriptase polymerase chain reaction (rRT-PCR), which is currently considered the most sensitive and specific SARS-CoV-2 test. Reverse-transcription loop-mediated isothermal amplification (RT-LAMP) tests have been proposed as a low-cost alternative to detect SARS-CoV-2 [1,2]. However, RT-LAMP tests have lower sensitivity than rRT-PCR tests, which can be a concern in high-risk settings, such as nursing homes. Similarly, SARS-CoV-2 antigen tests are fast and scalable point-of-care performance tests [3], but have lower sensitivity than most nucleic acid-based assays [4]. SARS-CoV-2 tests also include serological antibody response testing, which is not suitable for the diagnosis of acute infection, as antibodies can take several days or weeks to develop after viral infection. Therefore, serological testing is primarily utilized for the documentation of previous viral infections [5].

COVID-19 rRT-PCR kits are costly, thus making mass screening a burden for low-resource settings. Moreover, kit manufacturing can be hindered by deficiencies in required enzymes. Commonly available tests include COVID-19 rRT-PCR commercial kits and rRT-PCR COVID-19 assays developed by the World Health Organization (WHO) or Centers for Disease Control and Prevention (CDC) [6,7], which depend on commercial rRT-PCR Master mixes such as Thermofisher TaqPath™ 1-Step RT-qPCR Master Mix or Promega GoTaq**^®^** Probe 1- Step RT-qPCR System. The WHO test targets viral NSP12 (*RdRP*) and *E* genes, while the CDC test targets viral *N* genes; both protocols require amplifying each gene separately to prevent false positives. Hence, more reagents are consumed to diagnose each COVID-19 case. In this regard, formulating an in-house multiplex rRT-PCR master mix to detect at least two SARS-CoV-2 genes without false positive results represents a challenge and is necessary to reduce costs and overcome the shortage of reagents. Similarly, RNA extraction kits can also become a major bottleneck owing to limited supplies. In this respect, we report herein the development and validation of an in-house multiplex real-time RT-PCR diagnostic test for the detection of active COVID-19 infection (ScriptTaq COVID PCR). Furthermore, we describe two methods for RNA extraction using either in-house silica column or silica-coated magnetic beads, to replace commercial RNA extraction kits.

## 2. Materials and Methods

### 2.1. Establishment of an In-House Nucleic Acid Extraction Method

Bacterial expression vectors pUC57-2019-nCoV-PC: N and pUC57-2019-nCoV-PC: RdRP were purchased from Lucerna-Chem AG (Abendweg, Luzern, Switzerland), while RPP30 (NM_001104546) Human Tagged ORF Clone was obtained from Origene (Rockville, MD, USA). Following induction, the bacteria were processed for RNA extraction by several buffers to optimize the RNA extraction method. Two RNA purification methods were compared in this study: The silica-column-based method and magnetic-beads silica-coated based method. For the magnetic-bead silica-coated based method, different magnetic systems were evaluated. Biocomma magnetic beads (Cat # MSi100-0507-200 with a mean grain size of 100 nm and binding capacity of 12.5 µg/mg, 50 mg/mL) were used. SpiralPipet (M0022-S) Mix magnetic bead handling system, DynaMagTM-2 Magnet (ThermoFisher Scientific, Waltham, MA, USA), and Bio-Nobile QuickPick were compared in the magnetic-beads-based method. Invitrogen PureLink RNA mini kit (Invitrogen, Waltham, MA, USA) and QuickPick™ SML total RNA kit (Bio-Noble, Pargas, Finland) were used as reference methods.

The in-house RNA isolation kits established tested two lysis buffers and two wash buffers that would allow the collection of RNA only or combined RNA and DNA. Guanidine HCl, Guanidine Isothiocyanate, Tris, Tris-HCl, Nonidet P40, TWEEN 20, TCEP, Sodium Citrate, N-Lauroyl Sarcosine, Antifoam A, EDTA, ethanol, sodium acetate, NaCl, and nuclease-free water were all purchased from Sigma-Aldrich, Burlington, MA, USA. In-house magnetic beads and silica-column-based RNA isolation methods included the following buffers: Lysis Buffer I (7M Guanidine HCl, 50 mM Tris, 5% Nonidet P40, 2% TWEEN 20, 2 mM TCEP, pH 7.0) or Lysis Buffer II (3 M Guanidine Isothiocyanate, 1 mM TCEP, 10 mM Sodium Citrate, 0.5% N-Lauroyl Sarcosine, 0.002% Antifoam A, 100 mM Tris-HCl, 0.1 mM EDTA, 50% ethanol), Wash Buffer I (3 M sodium acetate pH 5.2), and Wash Buffer II (10 mM Tris, 1mM EDTA, pH 8, 70% ethanol). The in-house magnetic-beads-based nucleic acid (RNA/DNA) isolation method included the lysis buffer described above and Wash Buffer I (100 mM NaCl, 10 mM Tris/HCl, pH 7.5, 55% ethanol) and Wash Buffer II (100 mM NaCl, 10 mM Tris/HCl, pH 7.5, 70% ethanol). The elution buffer in all methods was nuclease-free water.

### 2.2. Nucleic Acid Extraction Using DynaMagTM-2 Magnet or QuicPick Magnetic Device

Lysis Buffer (300 μL) is added to the sample (200 μL) followed by 2.5 μL (40 mg/mL) proteinase K. The sample is vigorously vortexed for 15 s and incubated for 10 min at RT with intermittent vortexing. When Lysis Buffer I is used, an equivalent volume of 100% ethanol (500 μL) is added. This step is bypassed when Lysis Buffer II is used as it contains Ethanol. Then, 10 μL of magnetic beads is added, and the sample is gently vortexed and incubated for 5 min with continuous mixing. Magnetic beads are collected using a magnetic separator and the supernatant is removed. Wash Buffer I (500 μL) is added, and the sample is gently vortexed and incubated for 30 s with continuous mixing. Magnetic beads are then collected and the supernatant is removed. Wash Buffer II (500 μL) is added, and the sample is gently vortexed and incubated for 30 s with continuous mixing. Magnetic beads are then collected using the magnetic separator and the supernatant is removed. Magnetic beads are then air-dried and nucleic acid is eluted with 100 μL of nuclease-free water. Finally, magnetic beads are collected and the supernatant is transferred to a new tube. Nucleic Acid Extraction Using ABNOVA SpiralPipet and Autostage System reagents are added to the Auto Plate when using ABNOVA SpiralPipet and Autostage System, as indicated in Supplementary Table S1. The sample (300 µL) and 10 µL Proteinase K are added into column #1/ #7 of the Auto Plate. Auto Plate is placed onto the Autostage and ran according to the program indicated in Supplementary Table S2.

### 2.3. In-House RNA Isolation Kit Using Silica-Coated Magnetic Beads

Lysis Buffer (1000 μL) is added to the sample (200 μL) followed by vigorous vortexing for 15 s and incubated for 5 min at RT with intermittent vortexing. The lysed sample (700 μL) is then transferred to the spin cartridge and centrifuged for 15 s at 12,000× *g*. This is repeated until the entire sample has been processed. Wash Buffer I (700 μL) is added to the 2 mL silica spin cartridge (Cat # RP20, Biocomma Limited, Guangdong, China), followed by centrifugation for 15 s at 12,000× *g*. The spin cartridge is then washed twice with Wash Buffer II (500 μL). The spin cartridge is then centrifuged for 1–2 min at 12,000× *g*. Finally, RNA is eluted with 30–100 μL RNase-free water. Three sequential elutions of 100 μL each are performed when the RNA yield is expected to be more than 100 μg.

### 2.4. Establishment of In-House SARS-CoV2 Multiplex rRT-PCR Assay

WHO Coronavirus Charite protocol for COVID-19 has already been published [6]. This method served as a reference method for our ScriptTaq COVID PCR assay in addition to the CDC COVID-19 published method [7]. The COVID-19 rRT-PCR employed by the WHO and CDC utilizes NucleoSens easyMag system for RNA isolation and Invitrogen SuperScript III Platinum One Quantitative RT-PCR system (Life Technologies, Carlsbad, CA, USA). The DNA sequence of Taq Polymerase (GenBank: J04639.1) was artificially synthesized and cloned into pD454-SR bacterial expression vector by Atum.bio (Newark, CA, USA). Similarly, M-MLV Reverse Transcriptase (NCBI Reference Sequence: NP_057933.2) was artificially synthesized by Atum.bio and cloned into expression vector pD454-SR bacterial plasmid. The amino end of the protein contained a His-tag to simplify purification. In addition, the M-MLV RT gene had mutations (D524G, E562Q, and D583N) to eliminate RNase H activity and four mutations (H204R, M289L, T306K, and F309N) that together increased the half-life of M-MLV at 50 °C (expired patent: US12/861,797). The purified protein has an apparent molecular weight of 78 kDa. In addition, an aptamer (5′-CAAGACGGGCGGGTGTGGTAGGCGCCCGTG-3′) is added to the ScriptTaq COVID PCR assay (Noma et al., 2006). The aptamer inhibits ~80% of the Taq Polymerase activity. Expression plasmids (RdRP, N, and RPP30), Taq Polymerase, and the thermostable Reverse Transcriptase were transfected to the top 10 competent cells. Plasmids were transformed into the top 10 competent cells. Transformation was performed by thawing 50 µL of the top 10 competent bacteria and adding 10 ng of plasmid to pre-chilled tubes. The suspensions were kept on ice for 30 min, heated at 42 °C for 45 s for heat-shock, and then placed on ice again for 2 min. SOC media (250 µL) was added to the tubes and incubated in a shaker at 250 rpm for 1 h at 37 °C. Cells were streaked on antibiotic-resistant LB media plates and incubated at 37 °C overnight. Restriction mapping for the plasmids was performed using restriction enzymes HindIII and EcoRI (New England Biolabs (NEB), Ipswich, MA, USA). Plasmids were purified according to Qiagen Maxiprep Plasmid Kit protocol.

### 2.5. Taq Polymerase Purification

Two 2 L flasks containing 500 mL of Luria–Bertani broth media with 100 µg/mL ampicillin (LB/Ampicillin solution) are inoculated overnight with 15 mL of Taq polymerase-producing bacteria. The culture is induced by 0.5 mM IPTG (Isopropyl β-D-thiogalactopyranoside) once it reaches an optical density at 600 nm of 0.6. Cultures are centrifuged at 3500× *g* for 15 min and the pellet is resuspended in 25 mL of buffer A (50 mM of Tris-HCl, pH 7.9; 1 mM of EDTA, 50 mM of dextrose, 1 mM of PMSF). Resuspended pellets are frozen in liquid nitrogen and thawed at RT twice. Then, 1 mL of lysozyme (100 mg/mL) is added, followed by incubation for 15 min at RT. Then, 25 mL of lysis buffer (10 mM of Tris-HCl, pH 7.9; 1 mM of EDTA, 0.5% of Nonidet P40, 1 mM of DTT, 0.5% of Tween-20, 50 mM of KCl, 1 mM of PMSF) is added, followed by incubation for 30 min at 75 °C. The lysis mixture is centrifuged at 16,000× *g* for 10 min at 4 °C to remove the cell debris. To remove DNA and RNA, 500 µL of the digestive solution (10 mM of Tris-HCl, pH 7.5; 2 mM of CaCl2, 100 mM of MgCl2, 15 mM of NaCl, 50% of glycerol, 0.5 mg/mL of DNase Ι, 10 mg/mL of RNase A, 0.5 mM PMSF) is added and incubated for 5 min at RT. The mixture is incubated for 30 min at 75 °C to denature DNase Ι, followed by centrifugation at 16,000× *g* at 4 °C for 10 min. Ethanol is added to the supernatant to a final concentration of 55% followed by centrifugation at 16,000× *g* at 4 °C for 15 min. Finally, the pellet containing Taq Pol is dissolved in 10 mL of storage buffer (50 mM of Tris-HCl, pH 7.9; 1 mM of DTT, 50 mM of KCl, 0.1 mM of EDTA, 0.5 mM of PMSF, 50% of glycerol) and stored at −20 °C. Polymerase activity is determined as described previously [8]. The yield of Taq Polymerase enzyme was on average 45 mg/Liter of LB/Ampicillin Solution, with an average activity of 9550 units/mg protein, and with a total of 429,750 units/liter of the bacterial culture. 

### 2.6. His-Tagged Reverse Transcriptase Purification by TALON^®^ Chromatography

Cell lysates are prepared as described above. Reverse transcriptase-producing bacteria are induced by 0.5 mM IPTG overnight and the pellet is resuspended in 25 mL of buffer A (50 mM of Tris-HCl, pH 7.9; 50 mM of dextrose, 1 mM of EDTA, 1 mM of PMSF). Resuspended pellets are frozen in liquid nitrogen and thawed at RT twice. Then, 1 mL of Lysozyme (100 mg/mL) is added and the mixture is incubated at RT for 15 min. Then, 25 mL of lysis buffer (10 mM of Tris-HCl, pH 7.9; 1 mM of EDTA, 50 mM of KCl, 1 mM of DTT, 0.5% of Tween-20, 0.5% of Nonidet P40, 1 mM of PMSF) is added and the lysis mixture is incubated at RT for 30 min. A slurry of pre-swollen TALON**^®^** Superflow, supplied in 20% ethanol, is prepared by decanting ethanol solution and replacing it with distilled water at a ratio of 75% settled medium to 25% distilled water. The column is equilibrated with 5- to 10-bed volumes of binding buffer (50 mM sodium phosphate, 300 mM NaCl, pH 7.4) at a flow velocity of 150 cm/h. The column is then washed with wash buffer (50 mM sodium phosphate pH 7.4, 5 mM imidazole, and 300 mM NaCl) until the absorbance reaches the baseline. Reverse transcriptase is eluted with elution buffer (50 mM sodium phosphate, 300 mM NaCl, 150 mM imidazole, pH 7.4) using eight-bed volumes of elution buffer, which is then centrifuged in Amicon**^®^** Ultra-15 Centrifugal Filter (30 kDa). Thermostable reverse transcriptase was stored in a storage buffer (50 mM Tris-HCl pH 7.5, 300 mM KCl, 0.5 mM TCEP, 0.1% Triton X-100, 400 mM Trehalose, 5 mM MgSO4). A colorimetric Reverse Transcriptase Assay (Sigma Aldrich) is performed according to the manufacturer’s instructions. It avoids using [**^3^**H]- or [**^32^**P]-labeled nucleotides employed for the setup of the classical RT assay. The yield for thermally stable reverse transcriptase was on average 65 mg/Liter of LB/Ampicillin Solution, with an activity of 3450 units/mg protein, and with a total of 224,250 units/L of the bacterial culture.

### 2.7. Optimization of ScriptTaq COVID PCR Assay

The primers/probe sequences are listed in Table 1 and were synthesized by Bio Basic, Toronto, Canada. These primers/probes have been advised by the CDC and WHO [6,7]. A new probe for RPP30 had to be re-designed to give an NTC of 0 CT (Table 1), as the one advised by the CDC and WHO resulted in positive NTC when multiplexed with other primers/probes. rRT-PCR reactions were prepared by mixing 10 µL of 2X master mix (40 mM tris, pH 8.3, 100 mM KCl, 10 mM MgSO_4_, 1 M betaine, 0.4 mM dNTP, 0.5 mM TCEP, 0.02 U/µL RNase inhibitor, 0.125 U/µL Taq polymerase, 8 U/µL reverse transcriptase); primers/probe for RdRP, N, and RPP30 genes (F: 0.4 µM/ R: 0.4 µM/ Probe: 0.2 µM); and 5 µL nucleic acids processed sample. Tris, KCl, MgSO_4_, betaine, and TCEP were purchased from Sigma-Aldrich, while RNase inhibitor (RNasin) and dNTP were purchased from Promega (Madison, WI, USA). RNA isolated following IPTG induction of transformed *E. coli* with either pUC57-2019-nCoV-PC: RdRP, pUC57-2019-nCoV-PC: N, or RPP30 clones were used as positive controls. Fluorescence rRT-PCR detection was carried out on Biorad™ CFX96 Real-Time PCR Detection System with CFX Maestro Software (V. 2.3). Cycle parameters were set according to reverse transcription (50 °C, 10 min), cDNA pre-denaturation (94 °C, 2 min), and PCR cycling for 40 cycles (denaturation: 95 °C, 15 s; and annealing/extension: 60 °C, 45 s).

### 2.8. Statistical Analysis

Statistical analysis was carried out using SigmaStat software ver. 3.5 (Jandel Scientific, San Rafael, CA, USA). Significant changes in mRNA yields were computed for rRT-PCR results, and analysis was carried out using one-way ANOVA to compare the independent groups followed by (Holm–Sidak method) for pairwise comparisons. One-way ANOVA on Ranks was run followed by Dunn’s test for pairwise comparisons when normality distribution failed. *p*-value < 0.05 was used to assess the significance of all statistical analyses. The results are presented as mean ± standard deviation (SD).

## 3. Results

### 3.1. Development of In-House RNA Extraction Methods

**Figure 1:**
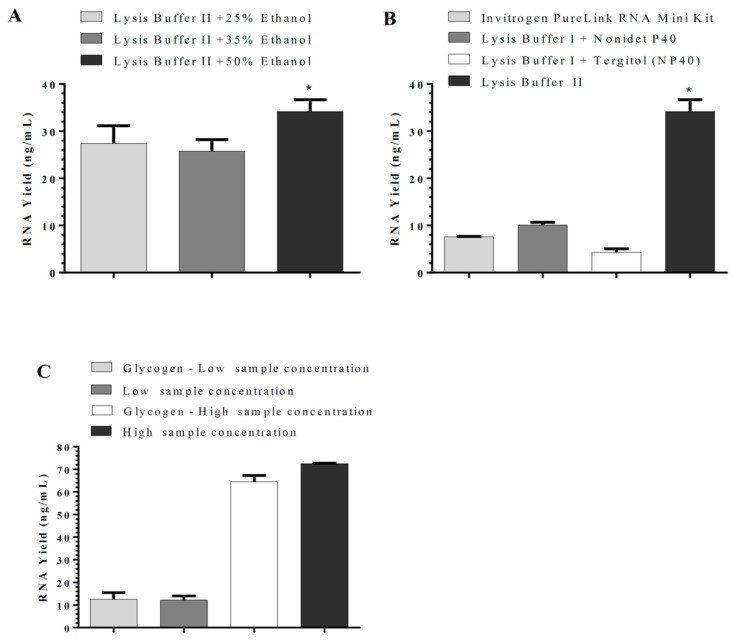
Optimization of lysis buffers’ ingredients used in our in-house silica-column-based RNA isolation kits; (**A**) effect of ethanol concentration on RNA binding to silica columns; (**B**) comparison of Invitrogen Purelink kit with the in-house RNA isolation in the presence of two non-ionic detergents (Nonidet P40 and Tergitol solution type NP-40); and (**C**) the effect of glycogen as carrier RNA on RNA yield using Lysis Buffer II and 50% ethanol. The results are presented as mean ± SD, *: *p <* 0.05 compared with other methods.

Bacterial expression vectors for *RdRP*, *N*, and *RPP30* genes were induced with IPTG to produce corresponding mRNAs. The bacteria following induction were processed for RNA extraction by several methods to optimize the RNA extraction method. Two RNA purification methods were compared in this study: The silica-column-based method and magnetic-beads silica-coated based approach. Tris(2-carboxyethyl) phosphine (TCEP) was utilized in our in-house RNA isolation. By modifications of the wash solution, either RNA or RNA/DNA were isolated in our in-house RNA isolation method. The effect of ethanol concentration on RNA binding to silica columns was determined using Lysis Buffer II. The ethanol concentration of 50% had the maximum yield compared with 25% and 35% (Figure 1A). The addition of non-ionic detergents (Nonidet P40 or Tergitol solution type NP-40) to Lysis Buffer I did not improve RNA yield (Figure 1B) using silica columns. Glycogen, as an RNA carrier, showed no significant effect on RNA yield when used with Lysis Buffer II (Figure 1C) at either high or low RNA levels.

**Figure 2:**
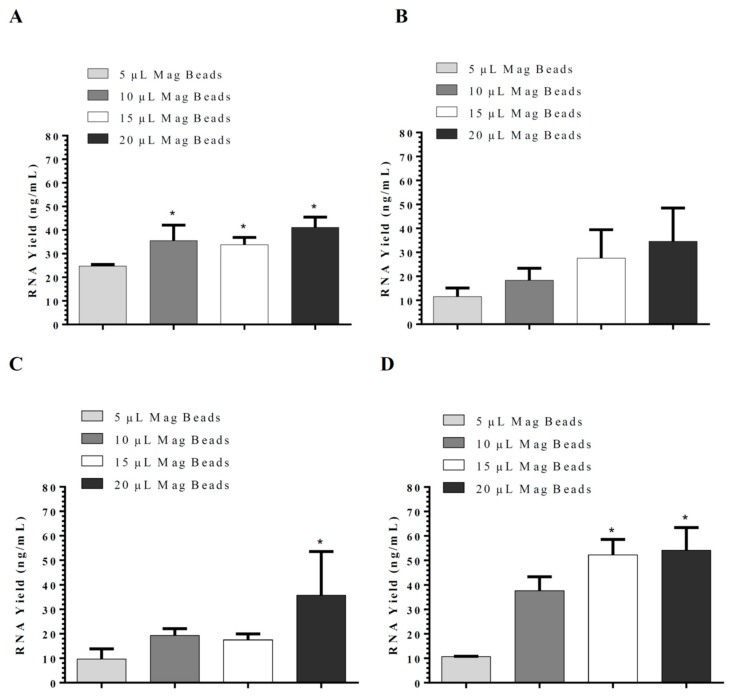
Optimization of lysis buffers used in our in-house RNA isolation kits utilizing silica-coated magnetic beads (50 mg/mL); (**A**) DynaMag^TM^-2 Magnet; (**B**) QuickPick; (**C**) SpiralPipet (M0022-S) Mix magnetic bead handling system; and (**D**) DynaMag^TM^-2 Magnet with 2.5 µL proteinase K (40 mg/mL). The results are presented as mean ± SD, *: *p <* 0.05 when compared with 5 µL volume.

**Figure 3:**
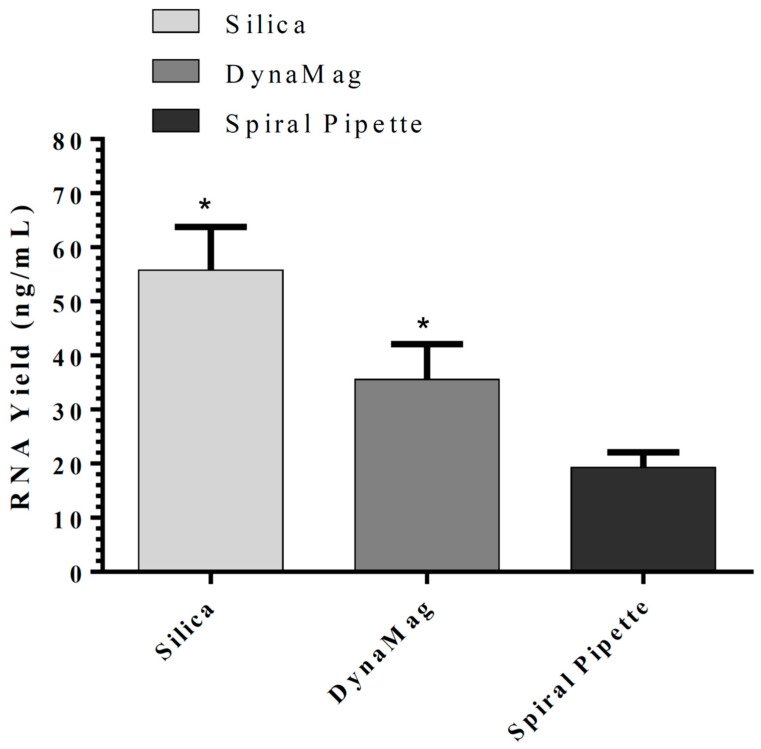
Comparison of the silica-based in-house RNA isolation kit and silica-coated magnetic beads (10 µL of 50 mg/mL) in-house RNA isolation method using either DynaMag^TM^-2 Magnet or SpiralPipet (M0022-S) Mix magnetic bead handling system. Similar buffers were used in the three methods for RNA isolation. The results are presented as mean ± SD, *: *p* < 0.05 when compared with other methods.

For the magnetic bead silica-coated based method, different magnetic systems were evaluated, including DynaMag^TM^-2 Magnet, SpiralPipet (M0022-S) Mix magnetic bead handling system, and Bio-Nobile QuickPick, which were compared in the magnetic-beads-based nucleic acid isolation methods. Utilization of DynaMag^TM^-2 Magnet resulted in maximum RNA yield (Figure 2) with a maximum binding obtained using 10 µL of 50 mg/mL Biocomma magnetic beads (mean grain size 100 nm), followed by QuickPick and SpiralPipet Mix magnetic bead handling system (Figure 2B,C). The addition of Proteinase K to the lysis buffer improved the RNA yield significantly when used with DynaMag^TM^-2 Magnet (Figure 2D). All methods described above had a coefficient of variation (CV) of less than 10% and RNA 260/280 nm absorbance ratios ≥1.8. Comparison of the silica-based in-house RNA isolation kit and silica-coated magnetic beads (50 mg/mL) in-house RNA isolation method using either DynaMag^TM^-2 Magnet or SpiralPipet (M0022-S) Mix magnetic bead handling system showed the best performance with silica-based spin columns (Figure 3). 

### 3.2. Development of the In-House RNA/DNA Extraction Method

**Figure 4:**
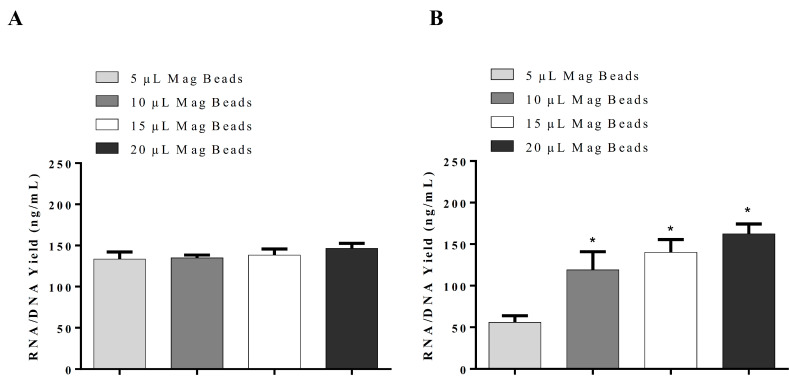
Optimization of lysis buffers used in our in-house RNA/DNA isolation kit with Wash Buffer I and II for the isolation of RNA and DNA utilizing magnetic beads (50 mg/mL); (**A**) DynaMag^TM^-2 Magnet and (**B**) SpiralPipet (M0022-S) Mix magnetic bead handling system. Proteinase K, 2.5 µL of 40 mg/mL, was added to Lysis Buffer II, and incubation was carried out at room temperature in both A and B. The results are presented as mean ± SD, *: *p* < 0.05 when compared with 5 µL volume.

**Figure 5:**
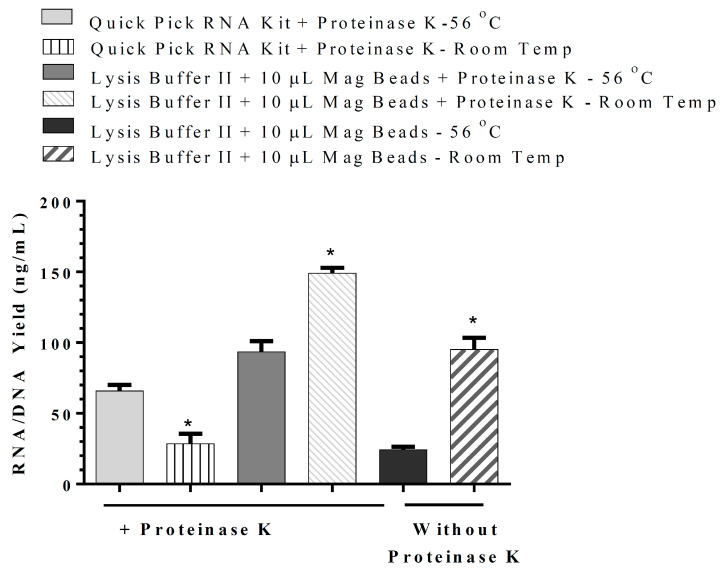
Comparison of our in-house RNA/DNA isolation kit with Wash Buffer I and II (RNA and DNA) utilizing silica-coated magnetic beads (50 mg/mL) with QuickPick™ SML total RNA purification kit, which requires incubation of the sample with Proteinase K—2.5 µL (40 mg/mL) at 56 °C. DynaMag^TM^-2 Magnets were used with both kits. The results are presented as mean ± SD, *: *p* < 0.05 when compared with the method at 56 °C incubation temperature.

By modifications of the wash solution, RNA/DNA were isolated in our in-house RNA/DNA isolation method using Wash Buffer I (100 mM NaCl, 10 mM Tris/HCl, pH 7.5, 55% ethanol) and Wash Buffer II (100 mM NaCl, 10 mM Tris/HCl, pH 7.5, 70% ethanol). Our goal was to develop a method that could isolate RNA/DNA to be used clinically for all DNA and RNA viruses. Treatment of the sample with DNase I would result in the collection of RNA only, similar to QuickPick™ SML total RNA purification kit. The results indicate that RNA/DNA isolation using the above-mentioned washing buffers would result in a high RNA/DNA yield with 10 µL of 50 mg/mL magnetic beads, even when the incubation of samples is carried out at RT (Figure 4). Comparison of our in-house RNA/DNA isolation method and QuickPick™ SML total RNA purification kit using DynaMag^TM^-2 Magnet, which requires incubation of the sample with Proteinase K at 56 °C, demonstrated the superiority of our in-house RNA/DNA isolation method at RT (Figure 5). 

### 3.3. ScriptTaq COVID PCR Assay

**Figure 6:**
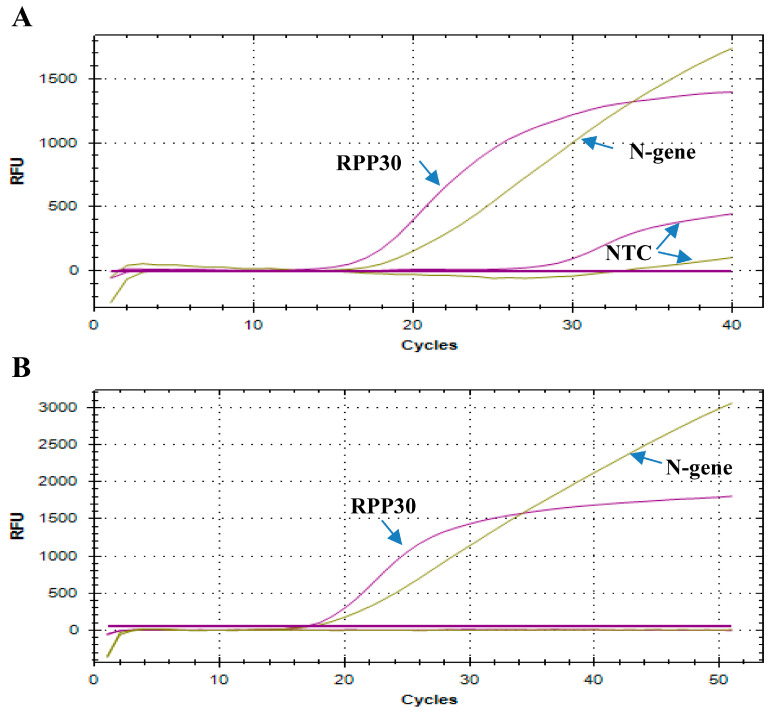
Effect of *RPP30* primers/probe designed for CDC protocol (*N1* and *RPP30*) on NTC. (**A**) Both (*N1* and *RPP30*) gave lower intensity and a high NTC in COVID-19 multiplex rRT-PCR; (**B**) the newly designed *RPP30* primers/probe gave no NTC up to 50 cycles and increased RFU.

The WHO assay targeted NSP12 (*RdRP*) gene and *E* gene, markers for SARS-CoV-2, and *RPP30* gene by rRT-PCR to monitor nucleic acid extraction efficiency and the presence of PCR inhibitors. On the other hand, the CDC COVID-19 assay can qualitatively detect the *N* gene for the new SARS-CoV-2 and the *RPP30* gene, which can confirm the validity of all test reactions. ScriptTaq COVID PCR assay design and validation strategy chose to exclude the *E* gene designed by Corman et al. [6] as it detects non-specific beta-coronavirus viruses. Several in-house PCR master mixes were evaluated for maximum sensitivity. Several PCR additives were tested to increase sensitivity. These additives included betaine monohydrate, Triton-X 100, polyethylene glycol (PEG) 8000, DTT (DL-Dithiothreitol; Clelands reagent), trehalose, and dimethyl sulfoxide (DMSO). The formulation of the multiplex rRT-PCR buffer with the highest sensitivity included is described in the Methodology section. One issue observed with the multiplex assay is a high NTC associated with the *RPP30* gene used in the CDC and WHO assays (Figure 6A). The primers/probe sequences are listed in Table 1. A new probe for *RPP30* had to be redesigned that gave an NTC of 0 CT (Table 1, Figure 6B). Studies were performed to determine the analytical limit of detection (LoD) for the in-house COVID-19 qPCR Multiplex Kit by determining the lowest concentration at which 19/20 replicates (95%) are positive for each SARS-CoV-2 RNA targets (true positive). The LoD for RdRP and N genes was 100 copies/PCR, as determined using Human 2019-nCoV RNA, Purified RNA of Coronavirus strain “BetaCoV/Germany/BavPat1/2020 p.1, obtained from European Virus Archive—GLOBAL (EVAg), Ref-SKU: 026N-03889”.

### 3.4. Validation of ScriptTaq COVID PCR Assay

#### 3.4.1. Inclusivity (Analytical Reactivity)

In silico analysis for the inclusivity of the used SARS-CoV-2 primer/probe was performed based on SARS-CoV-2 sequences from the NCBI database accessed on 14 February 2022. ScriptTaq COVID PCR assay is used for the qualitative detection of the NSP12 (RdRP) and N genes of SARS-CoV-2 RNA. Alignment analysis demonstrated 100% inclusivity for SARS-CoV-2 primer/probe sets for the RdRP gene and N gene. The alignment results for both genes are shown in Supplementary Table S3. According to in silico analysis, the primers/probes set in ScriptTaq COVID PCR assay can detect Alpha (B.1.1.7) variant (N501Y+HV69-70del), Beta (B.1.351) variant (E484K+K417N), Gamma (P.1) variant (E484K+K417T), Delta (B.1.617) variant (L452R+E484Q+P681R), and Omicron variant (B.1.1.529), as they showed 100% homology with these lineage sequences.

#### 3.4.2. Cross-Reactivity (Analytical Specificity)

No cross-reactivity was detected for the ScriptTaq COVID PCR assay when evaluated by both in silico analysis and by wet testing (Supplementary Table S4). The in silico mapping analysis of each primer/probe against 20 pathogens is based on the NCBI nr/nt database accessed on 14 February 2022, using the online Needle (EMBOSS) global alignment tool, and the results are shown in Supplementary Table S5. Cross-reactivity is observed when primers/probes all share homology >80% with the pathogen.

#### 3.4.3. Clinical Evaluation

The clinical performance of ScriptTaq COVID PCR assay (Table 2) was established using 240 oropharyngeal swab specimens from patients who were suspected of having COVID-19 and collected at two tertiary hospitals in Riyadh (100 patients from King Faisal Specialist Hospital (KFSH) and 140 patients from the Research Center at King Fahd Medical City (KFMC)). The comparator method at KFSH was CoDiagnostic Real-Time Fluorescent RT-PCR kit for Detecting SARS-2019-nCoV from CO-DIAGNOSTICS, INC. (2401 Foothill Dr., Ste D, Salt Lake City, UT 84109 USA) and that at KFMC was CareGENE-COVID-19 RT-PCR Kit (REF: MCD-N05082.MCD-N10082). The assay extraction method was Exiprep 96 viral DNA/RNA kit from Bioneer and both assays were run on Applied Biosystems ABI 7500 with SDS software version 1.5. An overall a positive percent agreement (PPA) of 90.2% and an overall negative percent agreement (NPA) of 100% were calculated. Both comparator methods, CoDiagnostic and CareGENE PCR Kits, are authorized for emergency use only. 

## 4. Discussion

Infection prevention and control measures are critical to combat the COVID-19 pandemic. The current testing utilizes either commercially available kits or the WHO- or CDC-developed COVID-19 assay, which requires commercially available PCR master mixes and RNA isolation kits. Commercially available coronavirus tests are currently available from different companies. However, these kits are expensive and the production of COVID-19 rRT-PCR kits is still slow and limited by shortages in the enzymes utilized in the manufactured kits. COVID-19 rRT-PCR assays also require viral RNA isolation kits and systems such as MagNA Pure Compact Nucleic Acid Isolation Kit I (Roche Applied Science, Penzberg, Germany) and NucliSENS easyMAG reagents and accessories (bioMérieux, Marcy-l’Étoile, France), which are also expensive. Thus, the adaptation of the in-house COVID-19 rRT-PCR assays would speed up testing and overcome the resource barrier in many world regions.

RNA isolation involves three steps: cell lysis, separation of unwanted products, and RNA elution. RNase activity must be inhibited throughout the procedure [9]. Many different protocols and variations have been published to optimize or simplify the RNA extraction process, with results comparable to those obtained using commercial RNA-extraction kits that can be used to detect SARS-CoV-2 by RT-qPCR [10]. In this study, an RNA extraction method with a silica spin column was evaluated as it is the most used method for RNA extraction. Many small laboratories still prefer it over the expensive methods utilizing beads. RNA binds specifically to the silica membrane in the spin column, while contaminants pass through it. PCR inhibitors are removed in the wash steps and pure RNA is eluted in either water or a buffer. The method established in this study showed superior efficiency to one of the commercially available kits. The Invitrogen PureLink RNA mini kit method uses β-mercaptoethanol or Dithiothreitol (DTT), which breaks the disulfide bond and loosens the secondary structure of RNA. However, β-mercaptoethanol and DTT are unstable in aqueous solutions and would limit our need for stability of reagents. Thus, TCEP was utilized in our in-house RNA isolation and was stable for up to six months at RT in the lysis buffer. Sodium lauroyl sarcosinate, also known as Sarkosyl, is an anionic surfactant derived from sarcosine and is used for solubilization and separation of membrane proteins and glycoproteins [11]. Sarkosyl was evaluated and used in our in-house lysis buffer for nucleic acid isolation instead of sodium dodecyl sulfate (SDS), which is commonly used in the commercial nucleic acid extraction kits and precipitates slowly below 16 °C. The use of Sarkosyl instead of SDS prevented precipitate formation usually observed in the nucleic acid isolation. By modifications of the wash solutions, only RNA was isolated in our in-house RNA or nucleic acids (RNA/DNA) isolation method. RNA extraction using silica-coated magnetic beads was also established to allow automation in the clinical laboratories. Glycogen, yeast tRNA, or any other RNA aid in the recovery of low-concentration RNA and can be removed during the 70% ethanol wash, thus reducing the chances of inhibition in downstream reactions. However, in our in-house RNA isolation method using lysis buffer II, glycogen had no significant effect on RNA yield. Moreover, using the same buffers with silica cartridges and silica-coated magnetic beads suggests that silica cartridges are more efficient than magnetic beads. The magnetic system used might affect the RNA yield as well.

The details of rRT-PCR assays for routine detection of SARS-CoV-2 have been published [6,7]. Detection of the *RPP30* gene is used as an internal control for sample collection and nucleic acid isolation method in the WHO and CDC assays. A high NTC associated with the *RPP30* gene primers/probes used was observed in both the CDC- and WHO-based assays with in-house multiplex PCR or commercially available PCR master mixes in our laboratory. A new probe for the *RPP30* gene had to be redesigned to give an NTC of 0 C_T_ in the multiplex PCR. The WHO assay targets the *RdRP* and *E* genes, while the CDC rRT-PCR assay target the SARS-CoV-2 nucleocapsid (N) protein gene and could be complemented by the *E* gene for screening and confirmation. Beta-coronavirus (β-CoVs or beta-CoVs) is one of four genera (alpha-, beta-, gamma-, and delta-) of coronaviruses. The beta-coronaviruses of the most significant importance concerning humans are OC43, and HKU1 of lineage A, SARS-CoV and SARS-CoV-2 of lineage B, and MERS-CoV of lineage C. Alignment analysis using EMBOSS NEEDLE for primers/probes for the *E* gene indicated high scores for SARS-CoV-2, SARS-CoV Tor2 strain, SARS coronavirus Urbani strain, and human coronavirus OC43 strain and HKU1 of lineage A. Thus, the *E* gene was excluded from the ScriptTaq COVID PCR assay as beta-coronaviruses could be amplified with the used primers/probes for the *E* gene.

Continuous mutations of the SARS-CoV-2 virus have been detected compared with Wuhan-Hu1 sequences or USA-WA1/2020, resulting in genetic variation in SARS-CoV-2 viral strains. This could potentially impact SARS-CoV-2 test performance. The FDA issued the Policy for Evaluating Impact of Viral Mutations on COVID-19 Tests [12]. To assess the mutation impact on the primers/probes utilized in our in-house COVID-19 rRT-PCR test, the locations of the primers/probes used were compared with all reported sequences for SARS-CoV-2 variants. Information regarding SARS-CoV-2 variants was obtained from BEI Resources, established by the National Institute of Allergy and Infectious Diseases (NIAID) [13]. The SARS-CoV-2 PCR primers/probes sequences for *N* and *NSP12 (RdRP1*) genes used had no reported mutations. Thus, the selected primers/probes could detect all SARS-CoV-2 variants reported until today. In addition, targeting two viral genes, *RdRP* and *N* genes, would lower SARS-CoV-2 PCR false negative results owing to SARS-CoV mutations in the regions detected by SARS-CoV-2 PCR primers/probes sequences. Thus, selection of target genes is critical and the use of two or more gene targets in the diagnostic assays is essential to maximize diagnostic accuracy (sensitivity and specificity). These key points emphasize the importance of the appropriate PCR primers/probes sequences’ design strategy to best capture SARS-CoV-2 variants and the need for the diagnostic assay quality.

The M-MLV RT (NCBI Reference Sequence: NP_057933.2) optimal temperature is 37 °C and thus would not provide a hot start capability for rRT-PCR. To overcome this limitation, the genetically modified clone for M-MLV RT was cloned and utilized in our in-house assay. The thermally stable Reverse Transcriptase was cloned with four mutations (H204R, M289L, T306K, and F309N) that together increased the half-life of M-MLV at 50 °C and thus allowed hot start rRT-PCR. In addition, to provide a better hot start for the PCR reaction, an affordable alternative to the *Taq* Polymerase antibody is used as the antibody is not available in large quantities and is expensive. To replace the *Taq* Polymerase antibody, an aptamer was tested to offer a cheaper and available alternative (5′-CAAGACGGGCGGGTGTGGTAGGCGCCCGTG-3′), as previously described [14]. The aptamer has an ~80% inactivation of *Taq* Polymerase.

TCEP is used in our in-house rRT-PCR master mix to replace DTT. DTT is a potent reducing agent widely used in molecular biology as an enzyme stabilizing agent by stabilizing proteins that possess free sulfhydryl groups [15,16]. Thus, its addition to the reverse transcriptase mix would protect the enzymatic activity of the reverse transcriptase. DTT addition also inhibits RNase activity by reducing the disulfide bonds in RNases [17]. DTT has also been shown to introduce nicks in the DNA backbone and to facilitate the immobilization of fluorescent DNA [18]. Adding TCEP to the PCR master mix resulted in comparable effects of DTT and offered stability to the PCR master mix as DTT is unstable in aqueous solutions.

Producing in-house rRT-PCR kits ensures a reduction in the expenses needed for the tests and makes them suitable for screening. Multiplexing also contributes to decreasing the expenses by testing multiple targets in one reaction in comparison with singleplex assays. Currently, there are many commercially available SARS-CoV-2 rRT-PCR kits available in the market. The prices range from $15 to $3 per test and the cost would increase substantially with shipping costs, on dry ice, taken into consideration. On the other hand, our in-house assay has an approximate cost of $0.2 per reaction. Nucleic acid extraction kits contribute to the cost of rRT-PCR; thus, an in-house RNA extraction procedure was established as well. Our in-house RNA extraction assay has an approximate cost of $0.5 per isolation. In conclusion, the in-house manufacturing of RNA isolation kits and rRT-PCR test with amplification enzymes, namely, thermostable reverse transcriptase, and *Taq* polymerase resulted in an extremely reduced cost and increased the ability to manage the limitation of supplies should any pandemic arise in the future.

In this study, ScriptTaq COVID PCR assay including kits for RNA isolation and multiplex rRT-PCR using in-house purified thermostable DNA polymerase and thermostable purified reverse transcriptase enzymes were established. Inclusivity of the primer/probe, RdRP gene, and N gene set used in the SARS-CoV-2 multiplex rRT-PCR assay was analyzed in silico and demonstrated 100% inclusivity for SARS-CoV-2 sequences and could detect all SARS-CoV-2 variants. Similarly, cross-reactivity of the SARS-CoV-2 multiplex rRT-PCR assay was evaluated by both in silico analysis and wet testing in clinical specimens. The in-silico mapping analysis of each primer/probe against 20 pathogens did not detect any cross-reactivity, while no cross reactivity was observed in clinical samples from patients with respiratory infections (MERS-coronavirus, Influenza A, Influenza B, and Rhinovirus). The method had an overall agreement of 95.4%, a PPA of 90.2%, and an NPA of 100.0% with two authorized for emergency use only kits at two different tertiary hospitals in Riyadh. However, validation of the test using FDA-approved kits is needed and by other laboratories to assess the inter-laboratory test variation. The utilization of ScriptTaq COVID PCR assay would reduce the costs substantially and would help to combat the COVID-19 pandemic, especially in low-resource settings. The platform established in this study could be adopted for other viruses and diseases.

## Figures and Tables

**Table 1:** Primer/probe sets verified and utilized by the CDC and WHO to test for COVID-19.

Gene	Sequences (5′→3′)	
RdRP gene	Forward	GTGAAATGGTCATGTGTGGCGG
	Probe	FAM-CAGGTGGAACCTCATCAGGAGATGC-BHQ1
	Reverse	CAAATGTTAAAAACACTATTAGCATA
N gene	Forward	CTGCAGATTTGGATGATTTCTCC
	Probe	VIC-5′-ATTGCAACAATCCATGAGCAGTGCTGACTC-3′-BHQ1
	Reverse	CCTTGTGTGGTCTGCATGAGTTTAG
Newly Designed RPP30 Sample Quality Control	Forward	AGATTTGGACCTGCGAGCG
	Probe	5′Cy5- TTCTGACCTGAAGGCTCTGCGC-BHQ-2
	Reverse	GAGCGGCTGTCTCCACAAGT

**Table 2:** Clinical evaluation between ScriptTaq, CoDiagnostic, and CareGENE PCR Methods. CoDiagnostic PCR Kit Comparator: positive percent agreement (PPA): 46/50 × 100% = 92.0%; negative percent agreement (NPA): 50/50 × 100% = 100.0%; overall agreement: 96/100 × 100 = 96.0%; CareGENE PCR Kit Comparator: PPA: 55/62 × 100% = 88.7%; NPA: 78/78 × 100% = 100.00%; overall agreement: 133/140 × 100 = 95.0%; Combined PCR Kit Comparators: PPA:101/112 × 100% = 90.2%; NPA: 128/128 × 100% = 100.0%; overall agreement: 229/240 × 100 = 95.4%.

Test		CoDiagnostic		CareGENE		Total
		Positive	Negative	Positive	Negative	
ScriptTaq	Positive	46	0	55	0	101
	Negative	4	50	7	78	139
Total		50	50	62	78	240

## Data Availability

Not applicable.

## Supplementary Materials

The following supporting information can be downloaded at https://www.mdpi.com/article/10.3390/cimb44120417/s1. Table S1: Auto Plate Content required for nucleic acid isolation when using ABNOVA SpiralPipet and Autostage System; Table S2: ABNOVA SpiralPipet and Autostage System program; Table S3: Representative results of in silico analysis for in-house COVID-19 Multiplex rRT-PCR against the reported 2019-nCoV Lineage sequences by 14 February 2022; Table S4: Cross-reactivity of in-house COVID-19 Multiplex rRT-PCR; Table S5: The in silico specificity analysis of primer and probe sets for other respiratory pathogens.

## References

[1] Jang W.S., Lim D.H., Yoon J., Kim A., Lim M., Nam J., Yanagihara R., Ryu S.W., Jung B.K., Ryoo N.H. (2021). Development of a multiplex Loop-Mediated Isothermal Amplification (LAMP) assay for on-site diagnosis of SARS CoV-2. PLoS ONE.

[2] Joung J., Ladha A., Saito M., Kim N.G., Woolley A.E., Segel M., Barretto R.P.J., Ranu A., Macrae R.K., Faure G. (2020). Detection of SARS-CoV-2 with SHERLOCK One-Pot Testing. N. Engl. J. Med..

[3] Sheridan C. (2020). Fast, portable tests come online to curb coronavirus pandemic. Nat. Biotechnol..

[4] Prendergast C., Papenburg J. (2013). Rapid antigen-based testing for respiratory syncytial virus: Moving diagnostics from bench to bedside?. Future Microbiol..

[5] Sethuraman N., Jeremiah S.S., Ryo A. (2020). Interpreting Diagnostic Tests for SARS-CoV-2. JAMA.

[6] Corman V.M., Landt O., Kaiser M., Molenkamp R., Meijer A., Chu D.K.W., Bleicker T., Brunink S., Schneider J., Schmidt M.L. (2020). Detection of 2019 novel coronavirus (2019-nCoV) by real-time RT-PCR. Eurosurveillance.

[7] Centers for Disease Control and Prevention, D.o.V.D. CDC 2019-Novel Coronavirus (2019-nCoV) Real-Time RT-PCR Diagnostic Panel. CDC/DDID/NCIRD/ Division of Viral Diseases, CDC-006-00019, Revision: 07. https://www.fda.gov/media/134922/download.

[8] Tveit H., Kristensen T. (2001). Fluorescence-based DNA polymerase assay. Anal. Biochem..

[9] Nilsen T.W. (2013). The fundamentals of RNA purification. Cold Spring Harb. Protoc..

[10] Wozniak A., Cerda A., Ibarra-Henriquez C., Sebastian V., Armijo G., Lamig L., Miranda C., Lagos M., Solari S., Guzman A.M. (2020). A simple RNA preparation method for SARS-CoV-2 detection by RT-qPCR. Sci. Rep..

[11] Frankel S., Sohn R., Leinwand L. (1991). The use of sarkosyl in generating soluble protein after bacterial expression. Proc. Natl. Acad. Sci. USA.

[12] U.S. Food and Drug Administration Policy for Evaluating Impact of Viral Mutations on COVID-19 Tests. https://www.fda.gov/regulatory-information/search-fda-guidance-documents/policy-evaluating-impact-viral-mutations-covid-19-tests.

[13] Biodefense and Emerging Infections Research Resources Repository (BEI Resources): SARS-CoV-2 Variant Table. https://www.beiresources.org.

[14] Noma T., Sode K., Ikebukuro K. (2006). Characterization and application of aptamers for Taq DNA polymerase selected using an evolution-mimicking algorithm. Biotechnol. Lett..

[15] Getz E.B., Xiao M., Chakrabarty T., Cooke R., Selvin P.R. (1999). A comparison between the sulfhydryl reductants tris(2-carboxyethyl)phosphine and dithiothreitol for use in protein biochemistry. Anal. Biochem..

[16] Netto L.E., Stadtman E.R. (1996). The iron-catalyzed oxidation of dithiothreitol is a biphasic process: Hydrogen peroxide is involved in the initiation of a free radical chain of reactions. Arch. Biochem. Biophys..

[17] Chen Z., Ling J., Gallie D.R. (2004). RNase activity requires formation of disulfide bonds and is regulated by the redox state. Plant Mol. Biol..

[18] Fjelstrup S., Andersen M.B., Thomsen J., Wang J., Stougaard M., Pedersen F.S., Ho Y.P., Hede M.S., Knudsen B.R. (2017). The Effects of Dithiothreitol on DNA. Sensors.

