# Supplementary material for: Development and Validation of ScriptTaq COVID PCR: An In-House Multiplex rRT-PCR for Low-Cost Detection
Source: Curr Issues Mol Biol. 2022 Dec 5;44(12):6117–31. doi: 10.3390/cimb44120417 (PMC9777119; doi:10.3390/cimb44120417)
Supplement: Supplementary file 1 [file cimb-44-00417-s001.zip › cimb-2023748-supplementary.pdf]

**Supplementary Table S1:** Auto Plate Content required for nucleic acid isolation when using ABNOVA SpiralPipet and Autostage System.

| Column | Buffer Solution | Volume |
|---|---|---|
| 1/7 | Lysis Buffer | 600 µL |
| 2/8 | Wash Buffer I | 800 µL |
| 3/9 | Wash Buffer II | 800 µL |
| 4/10 | Wash Buffer II | 800 µL |
| 5/11 | Magnetic Beads | 800 µL |
| 6/12 | Elution Buffer | 100 µL |

| Step | Well | Action | RPM | Time (Second) | CW/CCW (Second) | Temperature | Temperature Control |
|---|---|---|---|---|---|---|---|
| **1** | 5/11 | Collection | 0 | 30 | 0 | 60 | YES |
| **2** | 1/7 | Mixing | 3000 | 480 | 0 | 60 | YES |
| **3** | 1/7 | Collection | 0 | 30 | 0 | 60 | YES |
| **4** | 2/8 | Mixing | 3000 | 60 | 0 | 45 | YES |
| **5** | 2/8 | Collection | 0 | 30 | 0 | 45 | YES |
| **6** | 3/9 | Mixing | 3000 | 60 | 0 | 45 | YES |
| **7** | 3/9 | Collection | 0 | 30 | 0 | 45 | YES |
| **8** | 4/10 | Mixing | 3000 | 60 | 0 | 45 | YES |
| **9** | 4/10 | Collection | 0 | 30 | 0 | 45 | YES |
| **10** | 4/10 | Vapor | 0 | 300 | 0 | 45 | YES |
| **11** | 6/12 | Mixing | 3000 | 300 | 0 | 45 | YES |
| **12** | 6/12 | Collection | 0 | 30 | 0 | 45 | YES |
| **13** | 5/11 | Mixing | 3000 | 60 | 0 | 0 | NO |

**Supplementary Table S2:** ABNOVA SpiralPipet and Autostage System program.

| Strain | Lineage | Target | Accession | % Homology Test FP% | % Homology Test RP% | % Homology Test Probe% |
|---|---|---|---|---|---|---|
| hCoV-19/Scotland/CVR837/2020 | B.1.5 | N gene, RdRP | MT882022.1 | 100.0% | 100.0% | 100.0% |
| hCoV-19/Scotland/CVR2224/2020 | B.1.222 | N gene, RdRP | FR998185.1 | 100.0% | 100.0% | 100.0% |
| hCoV-19/Denmark/DCGC-3024/2020 | B.1.1.298 | N gene, RdRP | MT919525.1 | 100.0% | 100.0% | 100.0% |
| hCoV-19/Japan/TY7-503/2021 | P.1. (or 20J/501Y.V3) | N gene, RdRP | MZ252337.1 | 100.0% | 100.0% | 100.0% |
| hCoV-19/USA/MD-HP12112/2021 | B.1.1.207 | N gene, RdRP | MW049023.1 | 100.0% | 100.0% | 100.0% |
| hCoV-19/USA/MD-HP12155/2020 | B.1.1.4 | N gene, RdRP | FR995320.1 | 100.0% | 100.0% | 100.0% |
| hCoV-19/USA/MD-HP01101/2021 | B.1.1.7 | N gene, RdRP | MZ259007.1 | 100.0% | 100.0% | 100.0% |
| hCoV-19/USA/MD-HP01542/2021 | B.1.351 | N gene, RdRP | MW580574.1 | 100.0% | 100.0% | 100.0% |
| hCoV-19/USA/NY-NP-DOH1/2021 | B.1.526 | N gene, RdRP | MZ259007.1 | 100.0% | 100.0% | 100.0% |
| hCoV-19/USA/CA-Stanford-15_S02/2021 | B.1.617.1 | N gene, RdRP | MZ571142.1 | 100.0% | 100.0% | 100.0% |
| Severe acute respiratory syndrome coronavirus 2 TKYTK1734_2021 genomic RNA | B.1.617.2 | N gene, RdRP | LC633760.1 | 100.0% | 100.0% | 100.0% |
| hCoV-19/USA/NY-MSHSPSP-PV29369/2021 | B.1.1.1 (C.7) | N gene, RdRP | FR990203.1 | 100.0% | 100.0% | 100.0% |
| hCoV-19/USA/NY-MSHSPSP-PV28887/2021 | R.1 | N gene, RdRP, | BS001048.1 | 100.0% | 100.0% | 100.0% |
| BA.1 (omicron) | B.1.1.529 | N gene, RdRP | OL869974.1 | 100.0% | 100.0% | 100% |

**Supplementary Table S3:** Representative results of *In Silico* analysis for inhouse COVID-19 Multiplex rRT-PCR against the reported 2019-nCoV Lineage sequences by February 14, 2022.

| Virus | Source/ Sample type | Ct Value (RdRP gene/N Gene) |
|---|---|---|
| MERS-coronavirus (18 Samples) | Clinical specimen | Undetected |
| Influenza A (20 Samples) | Clinical specimen | Undetected |
| Influenza B (18 Samples) | Clinical specimen | Undetected |
| Rhinovirus (19 Samples) | Clinical specimen | Undetected |

**Supplementary Table S4:** Cross-Reactivity of inhouse COVID-19 Multiplex rRT-PCR.

| Pathogen (Taxonomy ID) | Strain | Target | GenBank Acc# | % Homology Test FP | % Homology Test RP | % Homology Test Probe |
|---|---|---|---|---|---|---|
| Human coronavirus 229E (11137) | camel/Abu Dhabi/B38 | N gene | MF593473.1 | 76.20% | 62.50% | 54.80% |
| Human coronavirus 229E (11137) | camel/Abu Dhabi/B38 | RdRP gene | MF593473.1 | 81.00% | 62.50% | 72.00% |
| Human coronavirus OC43 (31631) | HCoV_OC43/Seattle/USA/SC9 428/2018 | N gene | MN310476.1 | 86.40% | 67.94% | 83.30% |
| Human coronavirus OC43 (31631) | HCoV_OC43/Seattle/USA/SC9 428/2018 | RdRP gene | MN310476.1 | 86.40% | 76.90% | 45.80% |
| Human coronavirus HKU1 (290028) | HKU1 SC2628 | N gene | KY983584.1 | 70.00% | 45.50% | 70.80% |
| Human coronavirus HKU1 (290028) | HKU1 SC2628 | RdRP gene | KY983584.1 | 90.90% | 80.80% | 43.20% |
| Human coronavirus NL63 (277944) | HCoV_NL63/Seattle/USA/SC0 179/2018 | N gene | MN306018.1 | 70.00% | 43.20% | 70.80% |
| Human coronavirus NL63 (277944) | HCoV_NL63/Seattle/USA/SC0 179/2018 | RdRP gene | MN306018.1 | 82.60% | 88.00% | 72.00% |
| MERS-CoV (1335626) | BtVs-BetaCoV/SC2013 | N gene | KJ473821.1 | 75.00 | 58.30% | 55.90% |
| MERS-CoV (1335626) | BtVs-BetaCoV/SC2013 | RdRP gene | KJ473821.1 | 66.70% | 92.00% | 57.60% |
| Adenoviridae (10508) | 53/FS161/Fukui/2004 | N gene | AB568098.1 | 70.00% | 65.40% | 50.00% |
| Adenoviridae (10508) | 53/FS161/Fukui/2004 | RdRP gene | AB568098.1 | 82.60% | 65.60% | 73.10% |
| Adenoviridae (10508) | ITA/2018/251170-16 | N gene | MK625182.1 | 84.20% | 54.50% | 52.60% |
| Adenoviridae (10508) | ITA/2018/251170-16 | RdRP gene | MK625182.1 | 59.30% | 42.30% | 60.60% |
| Human metapneumovirus (162145) | bj0154 | N gene | MN745086.1 | 70.00% | 45.80% | 68.00% |
| Human metapneumovirus (162145) | bj0154 | RdRP gene | MN745086.1 | 37.20% | 56.20% | 50.00% |
| Human metapneumovirus (162145) | C-85473 | N gene | KM408077.1 | 60.90% | Not Sig | 43.60% |
| Human metapneumovirus (162145) | C-85473 | RdRP gene | KM408077.1 | 60.70% | Not Sig | 60.00% |
| Paramyxoviridae (11158) | MVs/Venezia.ITA/22.17/3[D8] | N gene | MK513627.1 | 77.80% | 69.60% | 61.30% |
| Paramyxoviridae (11158) | HPIV3/MEX/2822/2006 | RdRP gene | KF687324.1 | 71.40% | 65.40% | 54.30% |
| Orthomyxoviridae (11308) | A/Homo sapien/China/LS314/2019 | N gene | MT106847.1 | 76.20% | 67.90% | 63.00% |
| Influenza A virus (11320) | A/sanderling/New Jersey/756/1986 | RdRP gene | CY117434.1 | 56.70% | 57.70% | 37.70% |
| Orthomyxoviridae (11308) | A/sanderling/New Jersey/756/1986 | N gene | CY117434.1 | 77.30% | 64.00% | 54.80% |
| Influenza B virus (11520) | B/New York/20/2018 | N gene | MK999210.1 | 43.80% | 66.70% | 66.70% |
| Influenza B virus (11520) | B/Alabama/12/2019 | RdRP gene | MT029398.1 | 51.90% | 61.50% | 50.00% |
| Enterovirus (12059) | Donovan | N gene | AY421766.1 | 65.00% | 60.70% | 48.60% |
| Rhinovirus (12059) | Donovan | RdRP gene | AY421766.1 | 70.00% | 66.70% | 64.50% |
| Enterovirus (12059) | PS87/Belfast; ATCC VR-774 | RdRP gene | DQ092794.1 | 72.70% | 66.70% | 52.90% |
| Respiratory syncytial virus (12814) | B/WI/629-Q0306/10 | N gene | JN032121.2 | 68.20% | 68.20% | 59.30% |
| Respiratory syncytial virus (12814) | 99-901 | RdRP gene | MK947359.1 | 43.20% | 47.20% | 40.00% |
| Rhinovirus (12059) | PS-87 | RdRP gene | X79368.1 | 58.10% | 66.70% | 65.40% |
| SARSr-CoV (694009) | SARS coronavirus Frankfurt 1 | N gene | AB257344.1 | 69.60% | 91.70% | 95.70% |
| SARSr-CoV (694009) | SARS coronavirus Frankfurt 1 | RdRP gene | AB257344.1 | 95.50% | 96.20% | 88.00% |
| hCoV-19/pangolin/Guangxi/P4L/ 2017 | - | N gene | EPI_ISL_410 538 | 61.10% | 46.70% | 45.70% |
| hCoV- 19/pangolin/Guangxi/P4L/2017 | - | RdRP gene | EPI_ISL_410 538 | 45.50% | 69.20% | 65.20% |
| Bat coronavirus | | N gene | NC_048212.1 | 62.50% | 76.90% | 56.20% |
| Bat coronavirus | | RdRP gene | NC_048212.1 | 72.70% | 84.60% | 82.60% |

**Supplementary Table S5:** The *In Silico* Specificity Analysis of Primer and Probe Sets for Other Respiratory Pathogens.
